# 18S rRNA variability maps reveal three highly divergent, conserved motifs within Rotifera

**DOI:** 10.1186/s12862-021-01845-2

**Published:** 2021-06-10

**Authors:** Olaf R. P. Bininda-Emonds

**Affiliations:** AG Systematics and Evolutionary Biology, IBU–Faculty V, Carl von Ossietzky Universität Oldenburg, Carl von Ossietzky Strasse 9–11, 26111 Oldenburg, Germany

**Keywords:** Evolution, Phylogeny, Conservation, Hypervariable, Monogononta, Bdelloidea, Seisonacea, Acanthocephala, Deletion, Rate of evolution

## Abstract

**Background:**

18S rRNA is a major component of the small subunit of the eukaryotic ribosome and an important phylogenetic marker for many groups, often to the point of being the only marker available for some. A core structure across eukaryotes exists for this molecule that can help to inform about its evolution in different groups. Using an alignment of 18S rDNA for Rotifera as traditionally recognized (=Bdelloidea, Monogononta, and Seisonacea, but not Acanthocephala), I fitted sequences for three exemplar species (*Adineta vaga*, *Brachionus plicatilis*, and *Seison nebaliae*, respectively) to the core structure and used these maps to reveal patterns of evolution for the remainder of this diverse group of microscopic animals.

**Results:**

The obtained variability maps of the 18S rRNA molecule revealed a pattern of high diversity among the three major rotifer clades coupled with strong conservation within each of bdelloids and monogononts. A majority of individual sites (ca. 60%) were constant even across rotifers as a whole with variable sites showing only intermediate rates of evolution. Although the three structural maps each showed good agreement with the inferred core structure for eukaryotic 18S rRNA and so were highly similar to one another at the secondary and tertiary levels, the overall pattern is of three highly distinct, but conserved motifs within the group at the primary sequence level. A novel finding was that of a variably expressed deletion at the 3' end of the V3 hypervariable region among some bdelloid species that occasionally extended into and included the pseudoknot structure following this region as well as the central “square” of the 18S rRNA molecule. Compared to other groups, levels of variation and rates of evolution for 18S rRNA in Rotifera roughly matched those for Gastropoda and Acanthocephala, despite increasing evidence for the latter being a clade within Rotifera.

**Conclusions:**

The lack of comparative data for comparable groups makes interpretation of the results (i.e., very low variation within each of the three major rotifer clades, but high variation between them) and their potential novelty difficult. However, these findings in combination with the high morphological diversity within rotifers potentially help to explain why no clear consensus has been reached to date with regard to the phylogenetic relationships among the major groups.

**Supplementary Information:**

The online version contains supplementary material available at 10.1186/s12862-021-01845-2.

## Background

Together with numerous ribosomal proteins, 18S rRNA forms a major component of the small subunit of the eukaryotic ribosome. The single stranded RNA molecule itself has a characteristic and complicated secondary structure [see 1, 2], whereby it repeatedly folds back upon itself, with the resultant base-pairing or lack thereof creating stems and loops, respectively. Two or more stem-loop regions can also combine to form one of 14 different types of three-dimensional pseudoknot [3], thereby contributing to the tertiary structure of the molecule. In addition, eukaryotes share nine homologous regions in the molecule (and which are also present in the prokaryotic homologue 16S rRNA) that are especially variable and are labelled as the hypervariable regions V1—V9 [1]. (The eukaryotic V6 region, however, is noticeably less variable compared to the other regions [1] and to such a degree that it is not counted among the hypervariable regions by some authors (e.g., [4])). The molecule itself is encoded by the 18S rDNA gene, a distinction that I will maintain throughout this paper although the terms rRNA are rDNA are often used interchangeably in the literature.

The phylogenetic utility of 18S rDNA arguably stems in part historically from practical considerations. Together with 5.8S rDNA, 28S rDNA, and two internal and two external transcribed spacers, it forms an array that is tandemly repeated throughout the eukaryotic genome (e.g., ca. 300 + copies clustered across five chromosomes in humans [5]). The multicopy nature of the gene made it easier to extract and amplify in the pre-PCR era and concerted evolution meant that the many copies are virtually identical [6, 7], thereby sidestepping questions of paralogy. In addition, 18S rDNA as a gene is found universally among eukaryotes and possesses conserved flanking regions that facilitated primer design. However, its practical utility was augmented by the broad phylogenetic information content yielded by its structural characteristics, with the slower, more conserved stem regions (due to the constraints of the base pairing) providing resolution deeper in the tree to compliment the more recent information provided by the faster, less conserved loops and especially by the hypervariable regions. Indeed, much of the basis for deep phylogenies within Metazoa and beyond derive from phylogenetic analyses of 18S rDNA or other rDNA molecules more generally, both at the sequence and, more recently, the meta-sequence (i.e., structural) levels (e.g., [8–10]). At the other end of the spectrum, certain hypervariable regions, or parts thereof, have been promoted as possible species barcoding regions in diatoms (e.g., [11, 12]) and other “protists” (e.g., [13]). Although the early promise of 18S rDNA as “the” phylogenetic marker has not been realized, it remains one of the most widely sequenced genes across all organismal groups, especially in a phylogenetic context [14].

Indeed, 18S rDNA is often one of only a few, if not the only, phylogenetic marker sequenced for a given group. A case in point is Rotifera, a historically recognized phylum of approximately 2000 named species of microscopic animals [15] for which the only comprehensive molecular phylogeny to date encompasses 53 species (plus numerous outgroup species) sequenced for up to four markers including 18S rDNA as part of a total-evidence analysis with morphological characters [16]. Although countless studies based on either morphological or molecular data confirm the monophyly of the three major rotifer clades–Bdelloidea (ca. 388 species), Monogononta (ca. 1623 species), and Seisonacea (four species; more commonly referred to as Seisonidae)–their relationships to one another remain unclear [see 15], in part because of their highly distinct natures. Bdelloids are obligate asexuals (i.e., only parthenogenetic females are known) that can also undergo anhydrobiosis and whose genome has been shaped in part by (ancient) gene exchange. Monogononts, by contrast, are facultative asexuals that undergo cyclic parthenogenesis, possess only a single gonad and comprise many species presenting dwarf males to various degrees. Finally, seisonids are obligate sexuals with no male dwarfism and that live as ectoparasites or commensally on different species of the crustacean genus *Nebalia*, sometimes with different seisonid species living on different body parts of the same host [17, 18]. Even the application of molecular phylogenetics has failed to firmly resolve the relationships of these taxa to one another [15].

My goal in this paper is not so much to resolve the phylogenetic relationships within Rotifera, nor to address the question as to whether Acanthocephala nest within it (see “Methods”), but rather to examine rates of molecular evolution of the 18S rDNA gene within this traditional grouping based on newly obtained sequences combined with those present in GenBank. In so doing, however, my analyses reveal a pattern of highly distinct, yet conserved motifs among the major clades that might explain why there has been so little consensus about their interrelationships to date [see 15].

## Results

Examination of the uncorrected, average pairwise distances across groups determined using PAUP* 4.0a166 [19] revealed a pattern (Table 1A) whereby each of the taxonomic groups Bdelloidea, Monogononta, Seisonacea were all highly distinct from one another as well as from the platyhelminth outgroup *Calicophoron calicophorum* (> 14% divergence), but showed little internal variation (< 3%). This pattern is also reflected indirectly in the increase in the percentage of gaps in the Rotifera alignment compared to either of the Bdelloidea or Monogononta alignments (Table 2). In addition, sequences from monogonont species were, on average, slightly more similar to those of the outgroup than they were to the remaining rotifer clades. The use of corrected distances calculated using a GTR model of evolution revealed the same pattern (Table 1B), with average pairwise distances being on a par with (within clade comparison) or slightly higher than (between clade comparisons) the uncorrected distances.

**Table 1:** Average pairwise distances (A, uncorrected *p* distances ± SE; B, GTR distances ± SE) within (along the diagonal) and between (below the diagonal) the major rotifer clades determined using PAUP* v4.0a166 [19]

Taxon	Bdelloidea	Monogononta	Seisonacea
A			
Bdelloidea	0.0219 ± 0.0015 (595)		
Monogononta	0.1704 ± 0.0002 (5668)	0.0270 ± 0.0001 (13,039)	
Seisonacea	0.2226 ± 0.0011 (35)	0.1759 ± 0.0008 (162)	n/a (0)
* Calicophoron calicophorum*	0.2140 ± 0.0015 (35)	0.1406 ± 0.0006 (162)	0.2191 (1)
B			
Bdelloidea	0.0226 ± 0.0008 (595)		
Monogononta	0.1969 ± 0.0002 (5668)	0.0278 ± 0.0001 (13,039)	
Seisonacea	0.2692 ± 0.0016 (35)	0.2041 ± 0.0010 (162)	n/a (0)
* Calicophoron calicophorum*	0.2556 ± 0.0021 (35)	0.1568 ± 0.0007 (162)	0.2646 (1)

The number of pairwise comparisons is given in parentheses; it can be lower than the theoretical maximum of (*n*^2^ − *n*)/2 because of undefined distances (e.g., when the sequences of a species pair do not overlap). The corresponding average distances within all Rotifera were 0.0701 ± 0.0005 (uncorrected *p*) and 0.0787 ± 0.0006 (GTR) (each 19,499 comparisons) and between all Rotifera and *Calicophoron calicophorum* were 0.1539 ± 0.0021 (uncorrected *p*) and 0.1748 ± 0.0028 (GTR) (each 198 comparisons)

**Table 2:** Statistics relating to the different alignments used in this study

Data set	Aligned length	Percentage of gaps
Bdelloidea	1837	3.8% (2204 of 58,247 cells)
Monogononta	1868	3.2% (9077 / 286,203)
Rotifera	2021	10.8% (40,501 / 375,458)
Rotifera plus the outgroup *Calicophoron calicophorum*	2175	17.3% (70,540 / 407,479)

The percentage of gaps is corrected to ignore terminal gaps so as to better reflect the contribution of indels over potentially incomplete sequences

**Fig. 1:**
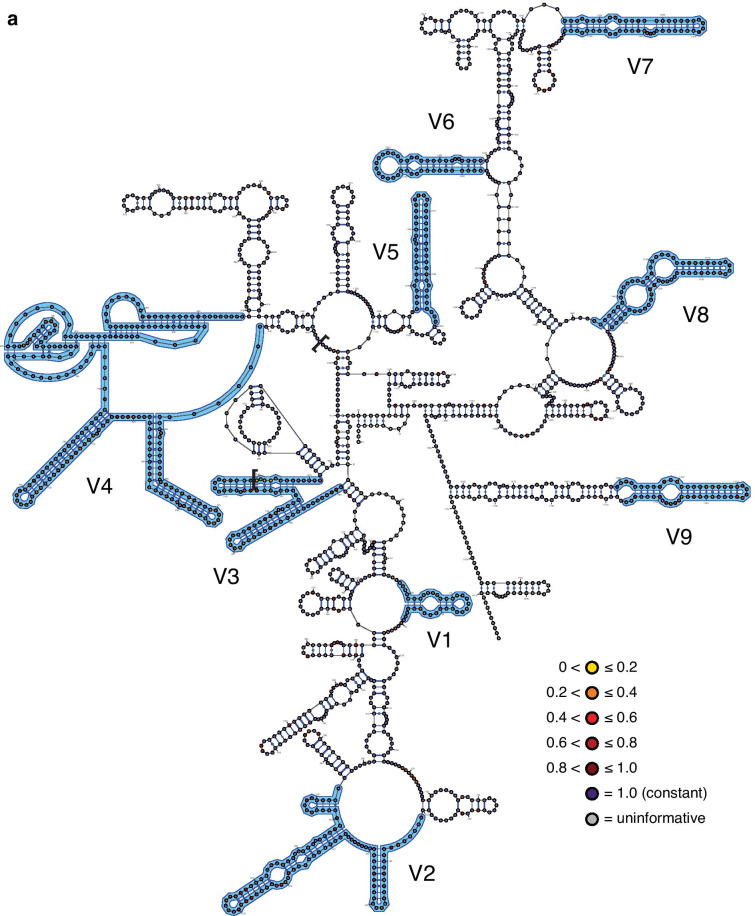

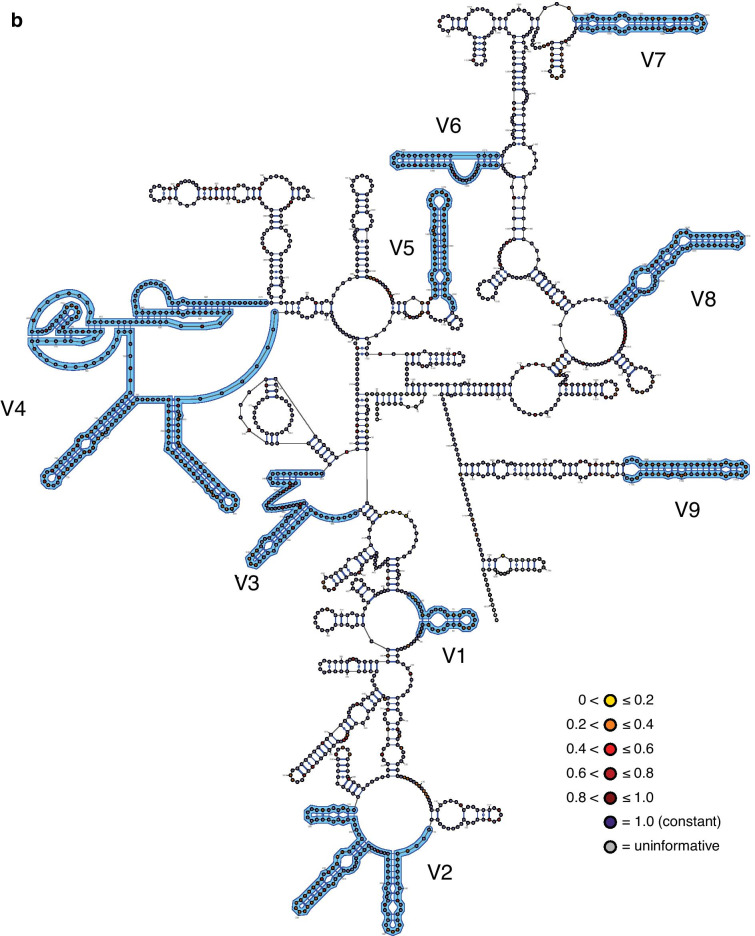

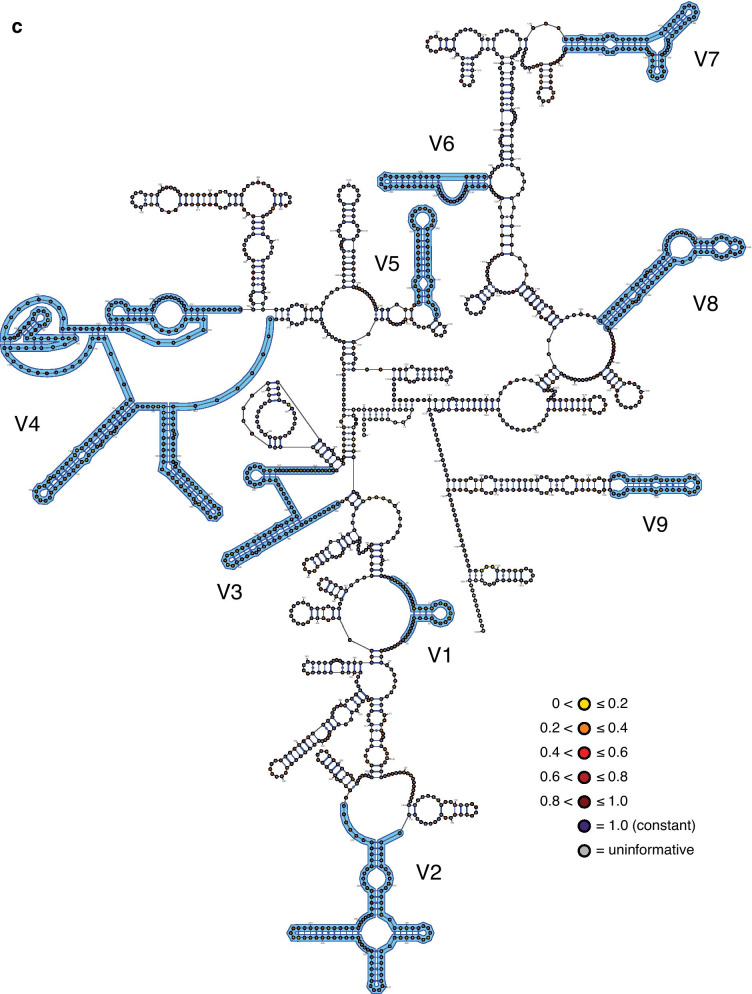
The three exemplar rotifer 18S rRNA molecules: **A** *Adineta vaga* (Bdelloidea), **b** *Brachionus plicatilis* (Monogononta), and **c** *Seison nebaliae* (Seisonacea). Individual nucleotides are coloured according to their relative rate of evolution (for Bdelloidea, Monogononta, and all Rotifera, respectively) as determined using TIGER (see inline legends) and hypervariable regions V1–V9 are labeled and outlined in blue. In addition, the maximal extent of the variable deletion at the 3'-end of the V3 region in bdelloids (see Fig. 2) is highlighted using square parentheses. Each map was created initially using VARNA v3.93 [59] and modified using Adobe® Illustrator® 2020 to match the traditional topology of the eukaryotic core structure as closely as possible. Empty circles were added at the terminal ends of the sequences as needed to present the presumed full length of the molecule for each species

**Fig. 2:**
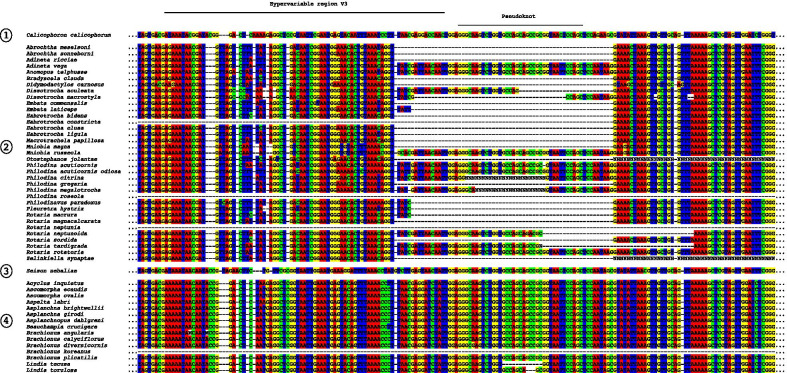
Partial alignment of the 18S rDNA gene showing the variably expressed deletion present at and extending beyond the 3' end of the V3 hypervariable region in bdelloids and two species in the monogonont genus *Lindia*. The locations of the entire V3 region as well as the pseudoknot following it are indicated at the top of the alignment. The taxonomic groups are numbered as (1) *Calicophoron calicophorum* (Platyhelminthes, outgroup), (2) Bdelloidea, (3) Seisonacea, and (4) Monogononta (selected species)

The inferred structural maps of the three exemplar rotifer species—*Adineta vaga* (Bdelloidea), *Brachionus plicatilis* (Monogononta), and *Seison nebaliae* (Seisonacea) (Fig. 1; see also Additional file 1)—each showed a good fit to the eukaryotic core structure for 18S rRNA proposed by Van de Peer and colleagues [20–22]. Unusual, however, is that most bdelloids (23 of 29 fully informative species; potentially the incompletely sequenced species *Otostephanos jolantae* and *Zelinkiella synaptae* as well) possess a unique and variably expressed deletion that spans 92 bps comprising the last 14 bps of region V3 and extends into the non-hypervariable region beyond this (Fig. 2). Six motifs of different lengths are present including the absence of the deletion and no species expresses the deletion in its full length (maximum deletion length is 68 bp). Although the deletion begins with a set of four nucleotides displaying very high relative rates of evolution (Fig. 1A), the patterns of the motifs suggest that the deletion originates from its 3′ end in a non-hypervariable region of the 18S rRNA molecule that is otherwise virtually constant in its sequence composition across Rotifera and encompasses most, if not all, of the pseudoknot following the V3 region in the eukaryotic core structure. The deletion is also variably expressed among species within each of the genera *Adineta*, *Embata*, *Mnobia*, *Philodina* and *Rotaria* (but not *Abrochtha*, *Dissotrocha*, or *Habrotrocha*) as well as in the species *Dissotrocha aculeata*, *Dissotrocha macrostyla*, *Philodina citrina*, and *Philodina megalotrocha*, with second GenBank sequences for each of these species (accession numbers JX494743, JX494745, JX494740, and JX494741, respectively) possessing even longer deletions. Altogether, this variability at both the genus and species levels together with the apparent presence of a more restricted deletion in the monogonont species *Lindia tecusa* and *Lindia torulosa* (Fig. 2) would suggest the convergent evolution of the deletion motifs barring any sequencing or identification errors for these GenBank sequences.

**Fig. 3:**
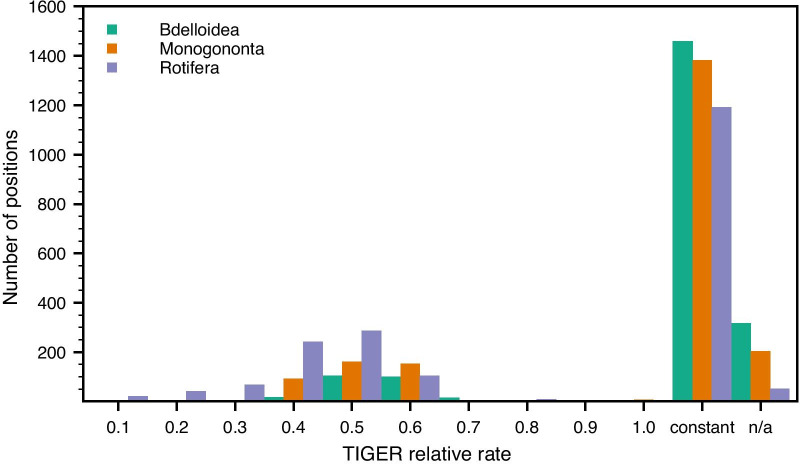
Histogram of relative TIGER rates of evolution across the three rotifer data sets (green, Bdelloidea; red, Monogononta; and blue, Rotifera). n/a stands for sites represented by less than 15% of the species in a given data set and so with insufficient coverage for the TIGER analyses

Average relative TIGER values across all sites were high, indicating slow rates of evolution (Table 3, Fig. 3), largely because > 60% of all sites (i.e., > 1000 bp) with sufficient coverage for the TIGER analyses within a given data set (Bdelloidea, Monogononta, and all Rotifera) were constant. The more homogenous bdelloid and monogonont data sets showed even greater proportions of constant sites (85.5% and 75.5%, respectively) and each also presented slower average relative rates across sites than did the entire rotifer data set. However, even the variable sites alone, which would include the hypervariable regions, were not unduly fast, with average TIGER values for Bdelloidea and Monogononta being slightly less than 0.5 and only decreasing to around 0.4 for all Rotifera (Table 3). Indeed, the rates for variable sites tended to cluster around these values such that variable sites that evolved extremely slowly (TIGER rate > 0.6) were all but absent as were those that evolved very rapidly (TIGER rate < 0.4) with the possible exception of across Rotifera as a whole.

**Table 3:** Relative TIGER rates of evolution across the three data sets for all sites and variable sites only, extended to include Acanthocephala

Data set	TIGER rate of evolution (all sites)	TIGER rate of evolution (variable sites)
Bdelloidea	0.926 ± 0.022 (n = 1703)	0.485 ± 0.031 (n = 244)
Monogononta	0.873 ± 0.020 (n = 1817)	0.467 ± 0.022 (n = 434)
Rotifera	0.760 ± 0.017 (n = 1969)	0.391 ± 0.014 (n = 777)
Acanthocephala	0.744 ± 0.015 (n = 2313)	0.371 ± 0.012 (n = 942)
Syndermata (= Acanthocephala + Rotifera)	0.661 ± 0.013 (n = 2480)	0.326 ± 0.009 (n = 1249)

Sites where less than 15% of the species possess sequence data are excluded. Rates are presented as the mean ± SE with no correction for the non-independence between paired sites

**Fig. 4:**
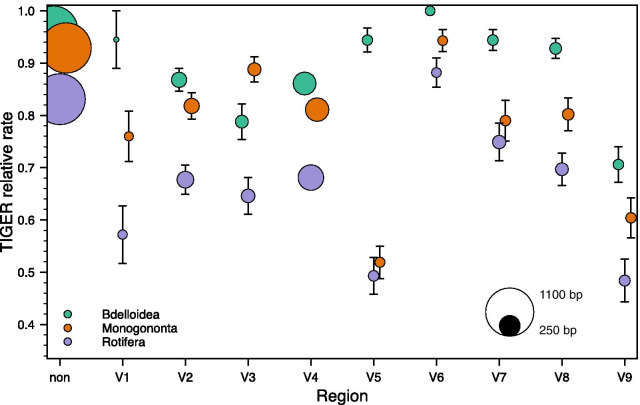
Sizes and relative rates of evolution (as determined using TIGER) of each of the hypervariable and pooled non-hypervariable regions for the three rotifer data sets (green, Bdelloidea; red, Monogononta; and blue, Rotifera). Error bars represent standard errors and are subsumed by the data point when not visible

There was no difference in the inferred relative rates of evolution between paired sites in each of the three data sets (column 2 in Table 4) and together these sites (“stems”) evolved slightly slower than unpaired sites (“loops”) for Bdelloidea only (column 3 in Table 4). Highly significant differences in relative rates were present between regions inferred as being within hypervariable versus non-hypervariable regions, regardless whether the former regions were pooled for the analysis or analysed individually (columns 4 to 6, respectively, in Table 4). The average relative rates for most hypervariable regions were faster than those for the pooled non-hypervariable regions (Fig. 4), with V6 being the notable, expected exception in addition to several hypervariable regions for bdelloids. In addition, the rates in the Rotifera data set were generally faster than those in either the bdelloid or monogonont data sets, reflecting in part the sequence differences between the motifs in the latter two. For the latter data sets, bdelloids generally presented slower rates than did monogononts with the exception of hypervariable region V3.

**Table 4:** Statistical summary for testing of potential differences in the relative TIGER rate of evolution between different categories of positions

Data set	Between paired positions	Stems vs. loops	Non-hypervariable vs. hypervariable regions – counts of constant vs. variable	Non-hypervariable vs. hypervariable regions – rates	Among hypervariable as well as pooled non-hypervariable regions
Bdelloidea	Wilcoxon *W* = 2211.5; *Z* = 1.055; *p* = 0.292; *n*_non-zero,total_ = 88, 437	Mann–Whitney *U* = 1.604 × 10^5^; *Z* = 2.969; *p* = 0.003; *n*_stems,loops_ = 443, 776	χ^2^ = 105.70, *df* = *1*; *p* = 8.60 × 10^–25^	Mann–Whitney *U* = 2.722 × 10^5^; *Z* = 10.608; *p* = 2.74 × 10^–26^; *n*_non,hyper_ = 1022, 663	Kruskal–Wallis *H* = 75.3; *p* = 2.12 × 10^–38^
Monogononta	Wilcoxon *W* = 5142.0; *Z* = 1.881; *p* = 0.060; *n*_non-zero,total_ = 131, 458	Mann–Whitney *U* = 1.827 × 10^5^; *Z* = 1.890; *p* = 0.059; *n*_stems,loops_ = 461, 834	χ^2^ = 162.95, *df* = 1; *p* = 2.57 × 10^–37^	Mann–Whitney *U* = 2.613 × 10^5^; *Z* = 13.217; *p* = 7.04 × 10^–40^; *n*_non,hyper_ = 1076, 683	Kruskal–Wallis *H* = 169.8; *p* = 6.92 × 10^–59^
Rotifera	Wilcoxon *W* = 11,856.0; *Z* = 1.0087; *p* = 0.313; *n*_non-zero,total_ = 209, 454	Mann–Whitney *U* = 1.866 × 10^5^; *Z* = 1.913; *p* = 0.056; *n*_stems,loops_ = 461, 859	χ^2^ = 130.18, *df* = 1; *p* = 3.74 × 10^–30^	Mann–Whitney *U* = 2.718 × 10^5^; *Z* = 11.190; *p* = 4.54 × 10^–29^; *n*_non,hyper_ = 1079, 701	Kruskal–Wallis *H* = 174.3; *p* = 2.03 × 10^–43^

Paired and unpaired positions were taken as proxies for stems and loops, respectively. Because paired rates were not significantly different from one another (column 2), each paired position was represented by only its single, average rate in column 3 to avoid problems with pseudoreplication. Only those positions that could definitely be assigned as being paired versus unpaired based on the structural map of the exemplar species (see Fig. 1) were included in the respective analyses

**Fig. 5:**
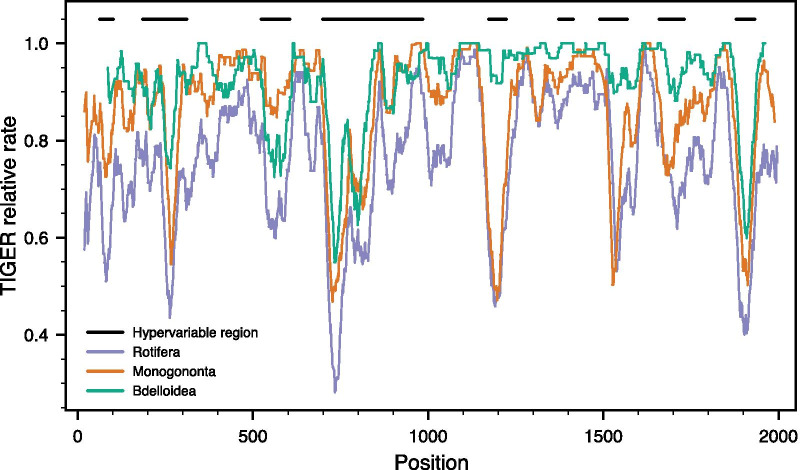
Relative TIGER rates of evolution presented as a rolling average of the 35 positions centred on the focal position for each of the three rotifer data sets (green, Bdelloidea; red, Monogononta; and blue, Rotifera). The locations of the hypervariable regions are indicated by bars at the top of the graph

Substitution hotspots were present along the entire molecule (Fig. 5), but more common and with higher relative rates in the hypervariable regions (with the exception of V6). However, most of these hotspots did not express unduly high relative rates of evolution. Relative rates within the hypervariable regions could also differ as exemplified by region V4, where the 5′ half of the region displays noticeably higher rates than the 3′ end where two pseudoknots are located. Again, rates of evolution at any given position were generally the highest for Rotifera and lowest for Bdelloidea among the three data sets.

Indels were numerous in each of the three exemplar species when aligned against one another in the Rotifera data set, and disproportionately so in the hypervariable regions, but were very short on average (usually < 2 bp long; Table 5). Only hypervariable region V7 showed a tendency toward having longer indels despite it being noticeably shorter in rotifers than in the outgroup species *C. calicophorum* (as well as in *Daphnia pulex*, and *Loricera foveata*; see Methods). Region V6 is noteworthy insofar as no indels were inferred between the three major rotifer clades (Table 5).

**Table 5:** Summary statistics regarding indels in each of the three exemplar rotifer 18S rRNA molecules from the complete Rotifera alignment (i.e., excluding *Calicophoron calicophorum*)

Region	Start position in the aligned rotifer data set	Length in the aligned rotifer data set	*Adineta vaga* (Bdelloidea)			*Brachionus plicatilis* (Monogononta)			*Seison nebaliae* (Seisonacea)		
			Number of nucleotides	Number of indels	Average length of indel	Number of nucleotides	Number of indels	Average length of indel	Number of nucleotides	Number of indels	Average length of indel
V1	64	39	33	4	1.5	33	3	2.0	35	3	1.3
V2	187	125	104	10	2.1	102	10	2.3	104	6	3.5
V3	524	83	74	7	1.3	75	6	1.3	77	4	1.5
V4	700	286	228	27	2.1	233	23	2.3	236	30	1.7
V5	1174	50	44	6	1.0	44	5	1.2	41	7	1.7
V6	1374	42	42	0	n/a	42	0	n/a	42	0	n/a
V7	1491	79	51	3	9.3	48	4	7.8	64	4	3.8
V8	1660	73	60	7	1.9	60	6	2.2	68	4	1.3
V9	1880	54	48	4	1.5	46	4	2.0	34	8	2.5
Non	n/a	1190	1135	50	1.1	1130	52	1.2	1129	51	1.2

For the pooled, non-hypervariable regions (“non”), terminal gaps, which likely represent incomplete sequences, were excluded from the calculations

## Discussion

Altogether, my examination of the evolution of 18S rDNA in rotifers revealed a clear pattern whereby the high morphological disparity among the three major clades is matched by their molecular disparity for this molecule. The high number of very short indels together with the extremely restricted sequence variation within each of the two largest clades (Bdelloidea and Monogononta) also indicates that the disparity derives mostly from substitutions, and then predominantly in the hypervariable regions. However, despite their name, even the hypervariable regions are largely conserved within the three major rotifer clades. By way of comparison, as well as to underscore the degree of sequence conservation within the major clades, the analogous average uncorrected pairwise distance for an alignment of 69 18S rDNA GenBank sequences for Acanthocephala (see Additional file 2) that includes all its four major subgroups within the clade is 13.1%. Although this value approaches that of the average pairwise divergence among the three rotifer 18S rDNA motifs (from 17.0 to 22.2% from Table 1 or 18.7% ± 1.5% among the three exemplar species only), it is an order of magnitude higher than that for within each of the major rotifer clades (< 3%; compare Table 1), a taxonomic status that arguably also applies for Acanthocephala (see Methods). Similarly, TIGER rates of evolution for Acanthocephala (Table 3) are comparable to those for Rotifera as a whole, rather than to those for either Bdelloidea or Monogononta.

**Fig. 6:**
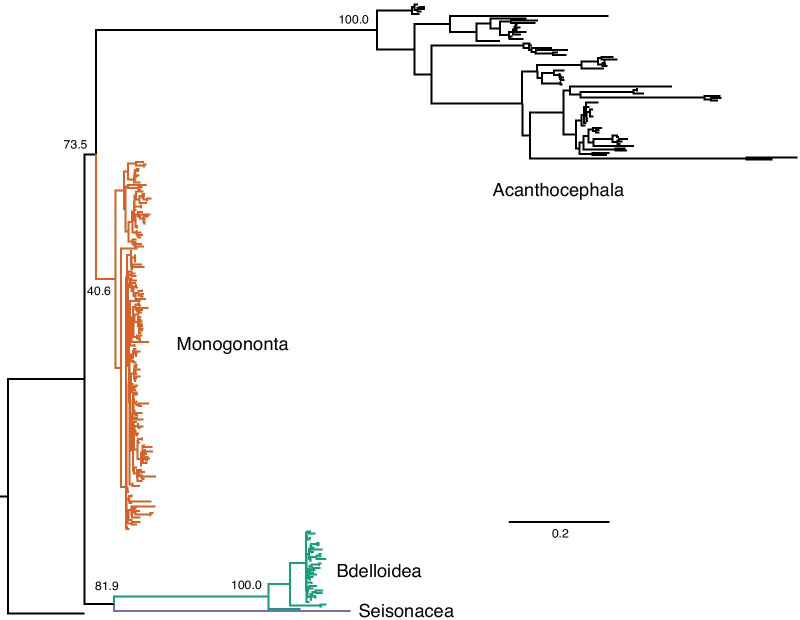
Maximum-likelihood phylogeny of the 18S rDNA sequences examined in this study using RAxML v8.2.12 [60]. The analysis consisted of a fast bootstrap search followed by a thorough search for the maximum-likelihood topology [61] under a GTR + Γ model, with the gamma distribution being approximated initially through a CAT model [62]. The tree was rooted on *Calicophoron calicophorum*. Values above selected nodes represent bootstrap support [63] and the scale bar represents the average number of substitutions per site per unit time. Species names have been removed for clarity and the major clades are labeled as well as colour-coded (green, Bdelloidea; red, Monogononta; blue, Seisonacea; black, Acanthocephala)

This extreme pattern in Rotifera is also easily visualized through a maximum-likelihood phylogeny of the sequences examined in this study together with those of Acanthocephala for context (Fig. 6; Additional file 3). Here it is the pattern that is of chief interest rather than the relationships per se, with Bdelloidea and Seisonacea forming sister taxa at the ends of extended branches to the exclusion of Monogononta, which shows much less divergence from the rotifer common ancestor. Monogononta also displays extremely reduced molecular divergence between its members, despite being by far the best represented of the three major clades. Finally, Acanthocephala again display as much internal molecular divergence in the phylogeny as for all true rotifers.

Moreover, the variability among the rotifer clades is restricted to at most 40% of the 18S rRNA molecule, with the remaining sites being constant across all Rotifera. This value coincidentally matches the proportion of the molecule that comprises hypervariable regions. However, even though the hypervariable regions are indeed significantly more variable than the non-hypervariable ones (both in the number of variable sites and their rates; Table 6), variable sites are found in both regions. Although the species sampling in this study was necessarily restricted, the taxonomic diversity of Rotifera was well represented insofar as exemplars from all major taxonomic subdivisions within each of Rotifera, Bdelloidea, and Monogononta (see Methods) were present in the data set. It could be argued that the number of constant sites is slightly overestimated insofar as gaps between the major clades were often preferred to substitutions. However, the increase in length compared to length of the entire molecular is negligible (about 10%; see Table 2) and this problem would apply chiefly to the entire rotifer data set. In addition, the high proportion of constant sites across Rotifera matches that inferred across Gastropoda by Weigand et al. [23] and for Acanthocephala (Table 3). Thus, the general patterns observed here, especially the highly distinct motifs for the major clades, are likely to be accurate. In addition, preliminary results from 28S rDNA and MT-CO1 confirm this general pattern of highly distinct motifs (results not shown), although not to the same extent as seen for 18S rDNA.

**Table 6:** Counts (with percentages in parentheses) of constant versus variable sites partitioned according to the inferred hypervariable versus non-hypervariable regions (pooled) of the three 18S rRNA data sets

Data set	Region	Counts		
		Total	Constant	Variable
Bdelloidea	Not hypervariable	1030 (60.5%)	940 (55.2%)	90 (5.3%)
	Hypervariable	673 (39.5%)	488 (28.7%)	185 (10.9%)
	All	1703 (100.0%)	1428 (83.9%)	275 (16.1%)
Monogononta	Not hypervariable	1111 (61.1%)	944 (52.0%)	167 (9.2%)
	Hypervariable	706 (38.9%)	411 (22.6%)	295 (16.2%)
	All	1817 (100.0%)	1355 (74.6%)	462 (25.4%)
Rotifera	Not hypervariable	1138 (57.8%)	784 (40.0%)	354 (18.0%)
	Hypervariable	831 (42.2%)	359 (18.2%)	472 (24.0%)
	All	1969 (100.0%)	1143 (58.0%)	826 (42.0%)

The results testing the hypotheses that the proportion of constant versus variable sites do not differ between regions of the 18S rRNA molecule is presented in Table 4 (middle column)

The lack of comparative data for other, comparable clades prohibits a good assessment of the novelty of the observed patterns across eukaryotes and the loss of readily available structural data through the demise of the European Ribosomal Database [24] is strongly felt in this regard. As mentioned, the high proportion of constant sites is comparable to that seen across Gastropoda, although the latter show a more even distribution of rates across the entire spectrum from slow to fast than was the case for Rotifera, albeit with a strong spike at intermediate rates [23]. Again, there is also a strong similarity in many parameters between Rotifera as a whole and Acanthocephala. Although the variability map of Van de Peer et al. [25] shows comparatively few constant sites and many very fast ones, it is at the level of all Eukaryota and so hardly comparable in terms of taxonomic breadth. The latter, however, does indicate that the pseudoknot following the V3 region is relatively conserved across eukaryotes, making its variable deletion within Bdelloidea all that more unusual. Even more extraordinary is that the deletion in both *Rotaria neptunoida* and *Rotaria tardigrada* includes the central “square” of the 18S rRNA molecule from which its three main arms originate and thereby could affect the tertiary structure of the entire molecule in some unknown and potentially severe fashion.

Regardless of the potential patterns in other taxonomic groups, one explanation for the pattern observed here is that the three rotifer crown groups are of relatively recent origin and, with the exception of Seisonacea, have undergone rapid adaptive radiations. If true, this scenario would imply a high degree of morphological plasticity in Monogononta in particular given both the higher number of species as well as morphological diversity across the group compared to Bdelloidea. What remains unclear, however, is why monogonont sequences, on average, remain more similar to that of a relatively distant platyhelminth outgroup (Table 1) instead of to the remaining rotifers or why this clade as a whole also does not subtend an extended branch like Bdelloidea and Seisonacea (and even Acanthocephala) as would be expected for a recent radiation within an otherwise ancient group. Of particular interest in this general context would be the sequencing of the remaining seisonid species. Given the lack of any obvious widespread dispersal abilities in seisonids, perhaps in concert with the hypothesis that they are among the oldest of the rotifer clades [17], the apparently exclusive association between them and their *Nebalia* hosts could be ancient, which agrees with the extended branch leading to *S. nebaliae*. Less clear, however, is whether the individual associations are also ancient or of more recent origin, especially given that different seisonid species can be found on the same host species if not the same host individual [17, 18]. Additionally, or alternatively, it could be that the three clades have independently reduced their rates of molecular evolution. However, it is not clear what the mechanism behind this would be and why the same process has not occurred in Acanthocephala, especially given that this taxon does indeed appear to nest within Rotifera [also 15, 16, 26–28]. Possible explanations for the latter discrepancy could lie with the endoparasitic lifestyle of all acanthocephalans as compared to the free-living true rotifers (with the exception of Seisonacea) or that the crown group is simply older and so shows more within-group molecular diversity.

Problematic in testing these hypotheses is that divergence time estimates for and within Rotifera are all but absent. Apart from a few reports of subfossilized rotifers from Holocene peat deposits (e.g., [29, 30]), the only other known rotifer fossils comprise contracted bdelloid specimens or their theca encased in amber, the oldest pieces of which have been dated to 35–40 Ma ago [31, 32]. Although the theca in particular have been assigned to the extant genus *Habrotrocha* [32], the contracted nature of the specimens and the simplistic features of the theca make it difficult to determine their species identity precisely and thus caution is perhaps advised as to whether or not either set of specimens belong to crown group Bdelloidea. Molecular based studies place the divergence between Rotifera and Platyhelminthes from anywhere between 492 to 1160 Ma ago (best estimate, 824 Ma ago; www.timetree.org [33]), with the origin of Rotifera being necessarily more recent than this, especially given that Platyhelminthes are likely not the immediate sister group of Rotifera, even among extant taxa [34, 35]. As such, it is unknown how old Rotifera are as a group as well as what the ages of its three major crown groups are, information that is needed to better understand the pattern of evolution of 18S rDNA witnessed in this paper, and possibly of other markers as well.

Nevertheless, the observed pattern potentially explains the severe problems in reconstructing the phylogenetic history of Rotifera at higher taxonomic levels. As summarized by Fontaneto and de Smet [15], about the only points of consensus in this context are the monophyly of each of the three major rotifer clades. Indeed, morphological and molecular analyses paint different pictures of higher-level rotifer phylogeny. Whereas the former tend to support Bdelloidea and Monogononta as sister taxa (= Eurotatoria) within a monophyletic Rotifera, the latter usually cluster Bdelloidea and Seisonacea together (as herein) within a paraphyletic Rotifera because of the inclusion of Acanthocephala, sometimes as the immediate sister group to Seisonacea. However, the pattern of evolution in 18S rDNA presented in this paper suggests that traditional sequence-based phylogenetic analyses of Rotifera could potentially be compromised by artefacts known to arise from long-branch attraction [see 36], which could be especially severe in this case given the lengths of the branches involved. In fact, the major rotifer clades are so distinct from one another molecularly that even analyses of MT-CO1 support the monophyly of each of Bdelloidea and Monogononta with high bootstrap support (results not shown), although MT-CO1 (or at least that part obtained using the Folmer [37] primers) normally loses phylogenetic signal above the genus level so rapidly that its use is typically restricted to species barcoding [38]. Instead, inferences based on rare genomic changes [see 39] or other types of markers, including meta-sequence features [see 40] might be more reliable for unravelling higher-level relationships within Rotifera. Interestingly, results from an analysis of mitochondrial gene order [28] do match those from traditional sequence analyses, despite the limited taxon sampling in that study as well as the methodological problems that are known for analyses of gene order and that have prevented these data from playing a more prominent role in phylogenetic analyses to date [see 41, 42].

Fortunately, any potential artefacts caused by long-branch attraction appear to be limited to the inferences of the relationships among the three major clades, rather than the relationships within each of them where the branches are shorter (see Fig. 6). At these less inclusive levels, the inferred relationships in the 18S rDNA tree (Additional file 3) tend to reflect accepted taxonomic groups down to the genus level within Rotifera, especially within Monogononta (see The Rotifera World Catalog (www.rotifera.hausdernatur.at), despite the high proportion of constant sites. As such, analogous to the case with missing data (see [43]), it would appear that the limited number of variable sites are both sufficient in number and do not evolve unduly rapidly to provide good resolution. Nevertheless, the rate variation among sites that is present requires that some correction for rate heterogeneity is used [see also 20, 23] and even this might be insufficient at higher taxonomic levels within Rotifera.

## Methods

### Data set

Largely complete 18S rDNA sequences (one per species; see Table S1, Additional file 2) were either compiled from GenBank or newly generated within the working group (87 sequences, all from Monogononta), in part for other studies (e.g., [44, 45]). All previously unpublished sequences have been deposited in GenBank under the accession numbers MT522624–MT522695 and MT542324. The newly derived sequences were obtained following the protocols outlined in Kimpel et al. [46] and Wilke et al. [45]. All taxonomic names were verified against The Rotifera World Catalog (accessed on April 10, 2020). In total, 198 rotifer sequences were used, including 162 monogonont sequences, 35 bdelloid sequences, and one seisonid sequence. Although this amounts to only roughly 10% of the described species diversity, all three major rotifer clades (Bdelloidea, Monogononta, and Seisonacea) were represented as were both major clades within Monogononta (Gnesiotrocha and Pseudotrocha; [15]) and all three within Bdelloidea (Adinetida, Philodinavida, and Philodinida; [47]); Seisonacea comprises only a single clade of four described species in two genera [18]. Although there is mounting molecular evidence that Acanthocephala (ca. 1330 species) nests within Rotifera, possibly as the sister taxon to Seisonacea (e.g., [16, 26–28]), I have restricted my primary analyses to “true” rotifers as traditionally recognized only. The complete 18S rRNA sequence for the flatworm *Calicophoron calicophorum* (Platyhelminthes: Trematoda: Digenea; GenBank accession L06566) was added to the data set as the most closely related outgroup to Rotifera for which a structural map was available (from the now defunct European Ribosomal Database; bioinformatics.psb.ugent.be/webtools/rRNA/secmodel/Ccal_SSU.html; [24]).

The data set was aligned initially using the default settings of MUSCLE v3.8.31 [48] as implemented in SeaView v5.0 [49] and then improved by eye to correct for obvious errors with reference to the structural map for *C*. *calicophorum* whenever possible. Automated alignment programs that account for secondary structure, including LocARNA [50], MAFFT [51], or RNAsalsa [52], could not be used because they cannot accommodate the pseudoknots present within the eukaryotic 18S rRNA tertiary structure (see Analyses below). The alignment, however, was relatively trivial insofar as areas of disagreement were usually localized to several discrete and obvious indels of various sizes, usually in the hypervariable regions. From the complete data set, two subsets comprising each of bdelloids and monogononts only were also constructed. The aligned length of the final data set (available as Additional file 4) and subsets of it (Bdelloidea, Monogononta, and all Rotifera including Seisonacea) can be found in Table 2.

### Analyses

Fitting the rotifer 18S rRNA molecules to the core structure of the eukaryotic 18S rRNA molecule proposed by Van de Peer and colleagues [20–22] is difficult because the latter contains three pseudoknots—one immediately following region V3 and two within region V4 [1, 2, 53]—as well as some other nested base pairings, each of which represent computationally hard problems [54, 55]. Although numerous programs exist that can compute pseudoknots or can fit a sequence to a given (partially resolved) structure based on minimizing free energies, none exist to my knowledge that can do both. In addition, it is likely unwise to attempt to constrain the structures of the hypervariable regions based on known structures from distantly related taxa as is the case here with the platyhelminth outgroup. Thus, to maintain the highest degree of accuracy as well as to incorporate as much information from the core structure as possible, I employed a two-step, ad hoc procedure to transfer the eukaryotic core structure to three representative rotifer rRNA sequences.

First, I obtained a conservative model of the core structure by aligning the complete 18S rRNA sequences for *C*. *calicophorum*, the water flea *Daphnia pulex* (Crustacea: Branchiopoda: Cladocera; GenBank accession AF014011), and the ground beetle *Loricera foveata* (Insecta: Coleoptera: Carabidae; AF012503), which together represent the most closely related outgroups to Rotifera for which structural maps are available from the European Ribosomal Database. The initial alignment again used MUSCLE, with the subsequent, manual refinement of it focussing exclusively on the more conserved, non-hypervariable regions with the aid of the structural maps. Again, for these relatively constant regions, the alignment procedure was relatively trivial. In the non-hypervariable regions, I used a conservative approach insofar as only those positions that were consistently paired versus unpaired across these three relatively distantly related reference sequences were constrained as such for the structural analyses of the rotifer sequences (denoted using paired parentheses and an x, respectively). The structure of all remaining positions was unconstrained (denoted using a period). All hypervariable regions as well as all pseudoknots and other nested pairings were constrained to be unpaired, thereby forming unresolved, extended loops initially. This generalized structural constraint was then applied to each of three rotifer exemplar species—*Adineta vaga* (for Bdelliodea), *Brachionus plicatilis* (for Monogononta), and *Seison nebaliae* (for all Rotifera)—using RNAfold v2.4.11 [56] to obtain the basic core structure. However, the constraint could be overruled by the program insofar as paired positions were not strictly enforced for these analyses (i.e., the switch—enforceConstraint was not set). Nucleotide positions unique to the rotifer sequences with respect to the core backbone (i.e., insertions) were left unconstrained (non-hypervariable regions) or constrained to be unpaired (hypervariable regions).

Second, the structures of the hypervariable regions in each exemplar rotifer species were resolved individually using RNAfold in the absence of any constraints apart from “bracketing” each region so that it began with a stem of at least two paired positions to provide some basal, structural context. Where possible, the brackets were obtained from the real sequences immediately adjacent to the hypervariable region according to the core backbone (regions V2, V4, V5, and V9). For regions V1, V6, V7, and V8, however, the brackets used obtained from the start of the hypervariable region, whereas an artificial bracket needed to be appended to either end of the sequences for region V3 and which was later removed. For region V4, only the 5′ region before the pseudoknots could be inferred in this fashion. The inferred structures for the hypervariable regions were then spliced into the core backbones determined in the previous step where these regions were forced to be unpaired and formed extended loops. Finally, the structures of the three pseudoknots and nested base pairings that RNAfold is unable to infer [56] were added by hand according to their homologous conserved positions and structures in the outgroup sequences.

Altogether, this procedure yielded structures that each strongly resembled the eukaryotic core structure and, within the context of the topological constraints, was based on an objective criterion for comparison (i.e., minimum free energies [56]) for those portions of the structure that were truly computed. The computational difficulties presented by the pseudoknots (e.g., RNAfold could not accurately estimate the structures of any of the three outgroup species in and around the regions of the pseudoknots, even given their own, complete structures as constraints) were also obviated in this manner.

Site-specific rates of molecular evolution were determined using the TIGER algorithm [57] as implemented in perlTiger.pl v1.0, with flanking gaps and Ns being ignored and ambiguous base calls being resolved into their component nucleotides. TIGER estimates relative rates of evolution using the congruence of site patterns between nucleotide positions as a proxy. The expectation is that slowly evolving positions will retain more phylogenetic signal and so, together with constant positions, will display more congruence with the remainder of the alignment. Conversely, positions that evolve rapidly should conflict with many others in the alignment. Thus, because TIGER determines relative rates in the absence of any phylogenetic information, it avoids some of the inherent circularity involved in determining absolute rates of evolution insofar as some a priori estimate of these rates (e.g., through a model of evolution) is needed to determine the molecular branch lengths from which the final rates are derived. Relative rates of evolution were determined for the entire data set as well as for the bdelloid and monogonont subsets of it. All analyses were restricted to the rotifer sequences (i.e., excluding *C. calicophorum*). Rates were only analysed for those sites where information was present for at least 15% of the species in the data set, a level chosen such that relative rates could still be calculated over the full rotifer data set for positions that comprised bdelloid sequences only (e.g., for bdelloid-specific insertions), but then still required at least 30 bdelloid species to be represented.

Non-parametric statistical analyses of the data were performed using the macOS version of PAST v4.02 [58] to test whether or not each of (1) inferred paired sites, (2) sites that were inferred to be in stems versus loops, and (3) non-hypervariable versus hypervariable sites evolved at the same relative rates. The last comparison was tested both between pooled hypervariable and non-hypervariable regions (Mann–Whitney U) as well as among all individual hypervariable regions and the pooled non-hypervariable regions (Kruskal–Wallis). The nominal alpha value for each test was 0.05 and no correction for multiple comparisons was used.

## Supplementary Information

**Additional file 1.** Fasta-formatted sequence file of 18S rDNA for the three exemplar rotifer species together with structural information in Vienna format.

**Additional file 2.** List of rotifer (Table S1) and acanthocephalan (Table S2) 18S rDNA sequences used in the study.

**Additional file 3.** Result files from the maximum-likelihood analysis of the alignment in Additional file 2.

**Additional file 4.** Fasta-formatted sequence alignment of 18S rDNA for all species examined in this study.

## Data Availability

The DNA sequence data supporting the results of this article are available in the GenBank® repository (http://www.ncbi.nlm.nih.gov) under accession numbers MT522624–MT522695 and MT542324 (see also Tables S1 and S2 in Additional file 2). perlTiger.pl is freely available at www.uol.de/systematik-evolutionsbiologie/programme. All other data generated or analysed for this study are included in this published article or its associated Additional files.

## Abbreviations

bp: Base pair
ca.: Circa
df: Degrees of freedom
DNA: Deoxyribonucleic acid
e.g.: For example
et al.: Et alia
Fig.: Figure
GTR: General time reversible
i.e.: In other words
Ma: Million years
macOS: Apple® Macintosh® operating system
MT-CO1: Mitochondrially encoded cytochrome c oxidase I
PAST: PAleontological STatistics
PAUP: Phylogenetic Analyses Using Parsimony
PCR: Polymerase chain reaction
RAxML: Randomized A(x)ccelerated Maximum Likelihood
rDNA: Ribosomal deoxyribonucleic acid
rRNA: Ribosomal ribonucleic acid
SE: Standard error
TIGER: Tree Independent Generation of Evolutionary Rates
VARNA: Visualization Applet for RNA
Γ: Gamma distribution for modeling rate heterogeneity
