# Correction: 18S rRNA variability maps reveal three highly divergent, conserved motifs within Rotifera

**DOI:** 10.1186/s12862-023-02128-8

**Published:** 2023-06-01

**Authors:** Olaf R. P. Bininda‑Emonds

**Affiliations:** AG Systematics and Evolutionary Biology, IBU–Faculty V, Carl von Ossietzky Universität Oldenburg, Carl von Ossietzky Strasse 9–11, 26111 Oldenburg, Germany

Correction: *BMC Ecol Evo ***21**, 118 (2021)

10.1186/s12862-021-01845-2

Following the publication of the original article [1], a potential error was confirmed that the author would like to inform the readers about.

In noting that many species of bdelloid rotifer display a shared, but variable-length deletion in the 18S rRNA molecule that could extend into a critical structural position (see original Figs. 1A and 2), it was also cautioned in the original article that these deletions could obtain from a sequencing artefact. The latter has now been positively confirmed by one of the authors of the sequences in question (Chris Wilson, pers. comm.) and the appropriate GenBank accessions will be amended to indicate this.
