# Supplementary material for: 18S rRNA variability maps reveal three highly divergent, conserved motifs within Rotifera
Source: BMC Ecol Evol. 2021 Jun 10;21:118. doi: 10.1186/s12862-021-01845-2 (PMC8194223; doi:10.1186/s12862-021-01845-2)
Supplement: Supplementary file 2 — Additional file 2. List of rotifer (Table S1) and acanthocephalan (Table S2) 18S rDNA sequences used in the study. [file 12862_2021_1845_MOESM2_ESM.docx]

**Table S1** Rotifer 18S rDNA sequences used in this study.

| Species | From GenBank | From the working group |
| --- | --- | --- |
| **Bdelloidea** |  |  |
| *Abrochtha meselsoni* | KM043249 |  |
| *Abrochtha sonneborni* | KM043250 |  |
| *Adineta ricciae* | KM043251 |  |
| *Adineta vaga* | GQ398061 |  |
| *Anomopus telphusae* | DQ089732 |  |
| *Bradyscela clauda* | KM043255 |  |
| *Didymodactylos carnosus* | KM043256 |  |
| *Dissotrocha aculeata* | KF561098 |  |
| *Dissotrocha macrostyla* | KF561099 |  |
| *Embata commensalis* | KM043257 |  |
| *Embata laticeps* | JX494742 |  |
| *Habrotrocha bidens* | KM043258 |  |
| *Habrotrocha constricta* | KM043259 |  |
| *Habrotrocha elusa* | KM043260 |  |
| *Habrotrocha ligula* | KM043261 |  |
| *Macrotrachela papillosa* | KM043262 |  |
| *Mniobia magna* | KM043263 |  |
| *Mniobia russeola* | AJ487049 |  |
| *Otostephanos jolantae* | KM043265 |  |
| *Philodina acuticornis* | U41281 |  |
| *Philodina acuticornis odiosa* | KY829026 |  |
| *Philodina citrina* | KF561100 |  |
| *Philodina gregaria* | KM043266 |  |
| *Philodina megalotrocha* | KF561102 |  |
| *Philodina roseola* | EF485012 |  |
| *Philodinavus paradoxus* | JX494731 |  |
| *Pleuretra hystrix* | JX494746 |  |
| *Rotaria macrura* | JX494738 |  |
| *Rotaria magnacalcarata* | KM043267 |  |
| *Rotaria neptunia* | AY218122 |  |
| *Rotaria neptunoida* | KF561103 |  |
| *Rotaria rotatoria* | DQ089736 |  |
| *Rotaria sordida* | KM043269 |  |
| *Rotaria tardigrada* | KF561105 |  |
| *Zelinkiella synaptae* | KM043271 |  |
|  |  |  |
| **Seisonacea** |  |  |
| *Seison nebaliae* | DQ089737 |  |
|  |  |  |
| **Monogononta** |  |  |
| *Acyclus inquietus* | KM873587 |  |
| *Ascomorpha ecaudis* |  | MT522624 |
| *Ascomorpha ovalis* | DQ297691 |  |
| *Aspelta labri* |  | MT522625 |
| *Aspelta secreta* |  | MT522626 |
| *Asplanchna brightwellii* | MK106105 |  |
| *Asplanchna girodi* |  | MT522627 |
| *Asplanchna sieboldii* | AF092434 |  |
| *Asplanchnopus dahlgreni* | DQ079916 |  |
| *Beauchampia crucigere* | KM873588 |  |
| *Brachionus angularis* | MK564749 |  |
| *Brachionus calyciflorus* | KF141790 |  |
| *Brachionus diversicornis* | MK106113 |  |
| *Brachionus koreanus* | KU314660 |  |
| *Brachionus plicatilis* | U49911 |  |
| *Brachionus quadridentatus* |  | MT522628 |
| *Brachionus rubens* |  | MT522629 |
| *Brachionus urceolaris* | DQ089734 |  |
| *Brachionus zahniseri* |  | MT522630 |
| *Bryceella stylata* |  | MT522631 |
| *Cephalodella forficula* | DQ297693 |  |
| *Cephalodella gibba* | AY218114 |  |
| *Collotheca campanulata* | DQ297686 |  |
| *Collotheca mutabilis* |  | MT522632 |
| *Collotheca ornata* |  | MT522633 |
| *Collotheca pelagica* |  | MT522634 |
| *Collotheca tenuilobata* | KM873589 |  |
| *Colurella adriatica* |  | MT522635 |
| *Colurella colurus* |  | MT522636 |
| *Colurella obtusa* |  | MT522637 |
| *Colurella uncinata* |  | MT522638 |
| *Conochilus coenobasis* | KM873590 |  |
| *Conochilus hippocrepis* |  | MT522639 |
| *Conochilus natans* |  | MT522640 |
| *Conochilus unicornis* | KM873591 |  |
| *Dicranophorus forcipatus* | DQ297694 |  |
| *Dicranophorus grandis* |  | MT522641 |
| *Dicranophorus luetkeni* |  | MT522642 |
| *Encentrum astridae* | DQ297695 |  |
| *Encentrum fluviatile* |  | MT522643 |
| *Encentrum marinum* |  | MT522644 |
| *Encentrum semiplicatum* |  | MT522645 |
| *Encentrum tectipes* | DQ297696 |  |
| *Eothinia elongata* | DQ079917 |  |
| *Epiphanes senta* | DQ089735 |  |
| *Euchlanis alata* | DQ079915 |  |
| *Euchlanis dilatata* | AY218116 |  |
| *Euchlanis oropha* |  | MT522646 |
| *Euchlanis triquetra* |  | MT522647 |
| *Filinia longiseta* | DQ079914 |  |
| *Filinia terminalis* | MK352482 |  |
| *Floscularia armata* | KM873592 |  |
| *Floscularia bifida* | KM873593 |  |
| *Floscularia decora* | AJ487047 |  |
| *Floscularia melicerta* |  | MT522648 |
| *Gastropus stylifer* |  | MT522649 |
| *Harringia eupoda* |  | MT522650 |
| *Hexarthra intermedia brasiliensis* | KM873594 |  |
| *Itura aurita* |  | MT522651 |
| *Kellicottia bostoniensis* |  | MT522652 |
| *Kellicottia longispina* |  | MT522653 |
| *Keratella cochlearis* |  | MT522654 |
| *Keratella quadrata* | KX358062 |  |
| *Keratella ticinensis* |  | MT522655 |
| *Lacinularia flosculosa* | KM873595 |  |
| *Lacinularoides coloniensis* | KM873596 |  |
| *Lecane bulla* | DQ297698 |  |
| *Lecane elsa* | DQ297699 |  |
| *Lecane inermis* | KY859765 |  |
| *Lecane leontina* | DQ297700 |  |
| *Lecane ludwigii* |  | MT522656 |
| *Lecane tenuiseta* |  | MT522657 |
| *Lecane ungulata* | KM873597 |  |
| *Lepadella ovalis* |  | MT522658 |
| *Lepadella patella* | AY218117 |  |
| *Lepadella rhomboides* | DQ297702 |  |
| *Lepadella triptera* |  | MT522659 |
| *Limnias ceratophylli* | KM873598 |  |
| *Limnias melicerta* |  | MT522660 |
| *Lindia tecusa* | DQ297703 |  |
| *Lindia torulosa* | DQ297704 |  |
| *Lophocharis salpina* |  | MT522661 |
| *Macrochaetus collinsii* | DQ297705 |  |
| *Microcodon clavus* | DQ297706 |  |
| *Mikrocodides chlaena* |  | MT522662 |
| *Monommata longiseta* |  | MT522663 |
| *Monommata maculata* | DQ297707 |  |
| *Mytilina bisulcata* |  | MT522664 |
| *Mytilina brevispina* |  | MT522665 |
| *Mytilina mucronata* | DQ297708 |  |
| *Mytilina trigona* |  | MT522666 |
| *Mytilina ventralis* | DQ297709 |  |
| *Notholca acuminata* | AY218115 |  |
| *Notholca foliacea* |  | MT522667 |
| *Notholca liepetterseni* |  | MT522668 |
| *Notholca squamula* |  | MT522669 |
| *Notommata allantois* | DQ297710 |  |
| *Notommata cerberus* |  | MT522670 |
| *Notommata codonella* | DQ297711 |  |
| *Notommata copeus* |  | MT522671 |
| *Notommata glyphura* |  | MT522672 |
| *Notommata tripus* |  | MT522673 |
| *Octotrocha speciosa* | KM873600 |  |
| *Pentatrocha gigantea* | KM873601 |  |
| *Plationus patulus* | DQ297712 |  |
| *Platyias quadricornis* | DQ297713 |  |
| *Pleurotrocha robusta* |  | MT522674 |
| *Pleurotrocha sigmoidea* |  | MT522675 |
| *Ploesoma hudsoni* | DQ297714 |  |
| *Ploesoma triacantha* |  | MK896812 |
| *Ploesoma truncatum* | DQ297715 |  |
| *Polyarthra euryptera* |  | MT542324 |
| *Polyarthra remata* | DQ297716 |  |
| *Polyarthra vulgaris* |  | MK896808 |
| *Pompholyx sulcata* |  | MT522676 |
| *Proales daphnicola* |  | MT522677 |
| *Proales doliaris* | DQ297717 |  |
| *Proales fallaciosa* |  | MT522678 |
| *Proales reinhardti* | DQ297718 |  |
| *Proales similis* | DQ297719 |  |
| *Ptygura beauchampi* | KM873602 |  |
| *Ptygura libera* | DQ297689 |  |
| *Ptygura longicornis* | KM873603 |  |
| *Ptygura pilula* |  | MT522679 |
| *Ptygura stygis* |  | MT522680 |
| *Resticula anceps* |  | MT522681 |
| *Resticula gelida* |  | MT522682 |
| *Rhinoglena frontalis* |  | MT522683 |
| *Scaridium longicauda* | DQ297720 |  |
| *Sinantherina ariprepes* | DQ297690 |  |
| *Sinantherina semibullata* | KM873604 |  |
| *Sinantherina socialis* | AY210451 |  |
| *Squatinella bifurca* |  | MT522684 |
| *Squatinella lamellaris* |  | MT522685 |
| *Squatinella rostrum* |  | MT522686 |
| *Stephanoceros fimbriatus* |  | MT522687 |
| *Synchaeta baltica* |  | MK896813 |
| *Synchaeta grandis* |  | MK896817 |
| *Synchaeta grimpei* |  | MK896818 |
| *Synchaeta gyrina* |  | MK896820 |
| *Synchaeta hutchingsi* |  | MK896838 |
| *Synchaeta kitina* |  | MK896831 |
| *Synchaeta oblonga* |  | MH481725 |
| *Synchaeta pectinata* | KF561106 |  |
| *Synchaeta stylata* |  | MK896825 |
| *Synchaeta tremula* |  | KY751520 |
| *Synchaeta tremuloida* |  | KY751528 |
| *Synchaeta triophthalma* |  | MK896828 |
| *Synchaeta vorax* |  | MK896832 |
| *Taphrocampa selenura* |  | MT522688 |
| *Testudinella clypeata* | KF561108 |  |
| *Testudinella elliptica* |  | MT522689 |
| *Testudinella patina dendradena* | KM873607 |  |
| *Testudinella reflexa* |  | MT522690 |
| *Trichocerca elongata* | DQ297721 |  |
| *Trichocerca mucosa* |  | MT522691 |
| *Trichocerca pusilla* |  | MT522692 |
| *Trichocerca rattus* | DQ297722 |  |
| *Trichocerca similis* |  | MT522693 |
| *Trichocerca tenuior* | DQ297723 |  |
| *Trichotria tetractis* |  | MT522694 |
| *Wulfertia ornata* |  | MT522695 |

**Table S2** Acanthocephalan 18S rDNA sequences used in this study.

| Species | GenBank accession number |
| --- | --- |
|  |  |
| **Archiacanthocephala** |  |
| *Macracanthorhynchus ingens* | AF001844 |
| *Mediorhynchus gallinarum* | KC261354 |
| *Mediorhynchus grandis* | AF001843 |
| *Moniliformis moniliformis* | Z19562 |
| *Oligacanthorhynchus tortuosa* | AF064817 |
|  |  |
| **Eoacanthocephala** |  |
| *Floridosentis mugilis* | AF064811 |
| *Hebesoma violentum* | KF156881 |
| *Neoechinorhynchus beringianus* | KF156875 |
| *Neoechinorhynchus crassus* | AF001842 |
| *Neoechinorhynchus pseudemydis* | U41400 |
| *Neoechinorhynchus saginata* | AY830150 |
| *Neoechinorhynchus salmonis* | KF156878 |
| *Neoechinorhynchus simansularis* | KF156877 |
| *Neoechinorhynchus tumidus* | KF156876 |
| *Paratenuisentis ambiguus* | AF469414 |
| *Tenuisentis niloticus* | KT970471 |
|  |  |
| **Palaeacanthocephala** |  |
| *Acanthocephaloides propinquus* | AY830149 |
| *Acanthocephalus anguillae* | AF469413 |
| *Acanthocephalus dirus* | AY830151 |
| *Acanthocephalus lucii* | AY830152 |
| *Andracantha gravida* | EU267802 |
| *Arhythmorhynchus brevis* | AF064812 |
| *Arhythmorhynchus frassoni* | JX442165 |
| *Bolbosoma balaenae* | JQ040305 |
| *Bolbosoma caenoforme* | KF156879 |
| *Bolbosoma turbinella* | JX442166 |
| *Bolbosoma vasculosum* | JX014225 |
| *Centrorhynchus conspectus* | U41399 |
| *Centrorhynchus globirostris* | KM588206 |
| *Centrorhynchus microcephalus* | AF064813 |
| *Corynosoma australe* | JX442168 |
| *Corynosoma enhydri* | AF001837 |
| *Corynosoma magdaleni* | EU267803 |
| *Corynosoma obtuscens* | JX442169 |
| *Corynosoma strumosum* | EU267804 |
| *Corynosoma validum* | JX442170 |
| *Dentitruncus truttae* | JX460860 |
| *Echinorhynchus gadi* | U88335 |
| *Echinorhynchus truttae* | AY830156 |
| *Echinorhynchus veli* | KP260659 |
| *Filisoma bucerium* | AF064814 |
| *Filisoma rizalinum* | JX014229 |
| *Gorgorhynchoides bullocki* | AY830154 |
| *Gymnorhadinorhynchus decapteri* | KJ590123 |
| *Hexaglandula corynosoma* | EU267808 |
| *Ibirhynchus dimorpha* | GQ981436 |
| *Koronacantha mexicana* | AY830157 |
| *Koronacantha pectinaria* | AF092433 |
| *Leptorhynchoides thecatus* | AF001840 |
| *Plagiorhynchus cylindraceus* | AF001839 |
| *Polymorphus minutus* | EU267806 |
| *Polymorphus obtusus* | JX442172 |
| *Polymorphus trochus* | JX442173 |
| *Pomphorhynchus bulbocolli* | AF001841 |
| *Pomphorhynchus laevis* | JX014223 |
| *Pomphorhynchus tereticollis* | AY423347 |
| *Profilicollis altmani* | AF001838 |
| *Profilicollis botulus* | EU267805 |
| *Profilicollis bullocki* | JX442174 |
| *Pseudocorynosoma anatarium* | EU267801 |
| *Pseudocorynosoma constrictum* | EU267800 |
| *Pseudoleptorhynchoides lamothei* | EU090950 |
| *Rhadinorhynchus lintoni* | JX014224 |
| *Rhadinorhynchus pristis* | JX014226 |
| *Serrasentis nadakali* | KC291715 |
| *Serrasentis sagittifer* | JX014227 |
| *Southwellina hispida* | JX014228 |
| *Transvena annulospinosa* | AY830153 |
|  |  |
| **Polyacanthocephala** | AF388660 |
| *Polyacanthorhynchus caballeroi* | AF388660 |
